# 

**Published:** 1887-07-30

**Authors:** 


### The Hospital.

**An Institution, Family, and Parochial Journal of Hospitals, Asylums, and all Agencies for the Care of the Sick, Criticism and News.**

SATURDAY, JULY 30TH, 1887.

### Notice.

On and after August 1st, 1887, The Hospital will be published at 2, Paternoster Square, E.C. Mr. A. P. Watt will, from the date named, assume the business management of the Journal, and all communications relative to the Publishing and Advertising Departments should, after August 1st, be sent to him. All accounts due for advertising in The Hospital from April 1st, 1887, must be paid to Mr. A. P. Watt, whose receipt will be sufficient discharge.

### NOTICE TO CORRESPONDENTS.

All correspondence must be written on one side of the paper only.
We cannot undertake to return MSS. not used.

**Communications,** whether intended for insertion or for private information, must be authenticated by the names and addresses of the writers, not necessarily for publication.

**Local papers** containing reports or news paragraphs **should be marked.**

It is especially requested that early intelligence of **local events of interest,** or which it may be thought desirable to bring under notice, may be sent direct to the Editor.

Communications respecting editorial matters should be addressed to the Editor, Norfolk House, Norfolk Street, Strand, London, W.C.

### The Literary Prize.

A prize of **Two Guineas** will be given every month for the best story or incident based upon hospital, or asylum, or student life, or any incident connected with the professions of medicine or nursing.

⁂ The Children's Corner and Amusements with Profit will be found on page vi of this week's issue.

### Questions and Answers.

All questions should be addressed to Mr. Howard J. Collins, Secretary, The Hospitals Association, Norfolk House, Norfolk Street, Strand, W.C.

### COTTAGE HOSPITALS.

Can you inform me if there is a directory published containing a list of cottage hospitals? We are trying to erect and maintain one in this place, and would be glad to obtain particulars on both these points.—H. S. H.

*** Similar inquiries to the above are frequently made, and we take the opportunity of saying that full particulars will be found in Mr. Henry C. Burdett's book, entitled "Cottage Hospitals, their Origin, Construction, Management, and Work," published by Messrs. J. and A. Churchill.—[Ed. T. H.]

### HOME FOR A LADY.

Can you help me in finding a comfortable Home for a lady who is both physically and mentally weak? I have been recommended by Miss Vincent, of the "Home" at Reading, to apply to you. The lady in question is entirely dependent on me, and as I am a widow with ouly a small income, I cannot afford to pay more than £25 a year. She really gives little or no trouble, and her living, by the doctor's orders, is most plain and simple. She never takes stimulants, only medicinally, and that is very seldom. She has lived with me for several years, but now I find it is too great a tie to me. I shall be so much obliged if you can in any way help me.—S. C.

*** Answers may be addressed to the Secretary, Hospitals Association, Norfolk House, Norfolk Street, Strand, W.C.—[Ed. T. H.]

M. B. desires to know if there be any easy method of finding out vacancies for matrons, etc., at the hospitals as they arise.

*** A list of "Vacancies" is published in The Hospital every week.

### ANSWERS TO CORRESPONDENTS.

Miss Charlotte Catherine Bacon is thanked for her communication.

Miss Mary E. Helsby.—Your note with enclosure received. The Editor congratulates you on your decision. Pressure on space will not admit of publication at present.

Miss E. R. Miles.—Received. Shall have attention.

### The In- and Out-Patients' Hospital Diary.

This Diary is so arranged as to make some portion of it appear every week, and the whole in each monthly part. It has been very difficult to compile, and corrections will at all times be gratefully received. It has been suggested that similar information about the larger Provincial and Scotch Hospitals would be useful to many readers. We should be glad to hear if this is felt to be the case.

### DISEASES AND IRREGULARITIES OF THE TEETH.—*continued.*

| Hospitals and Terms of Admission. | Physicians, Surgeons, and Medical Officers, with Days and Hours of Attendance. |
|---|---|
| New Hospital for Women, 222, Marylebone rd., W. | *Surgeon*, Mr. H. Apperley |
| North-West London Hosp., 18, Kentish Town Rd., N.W. | *Surgeon*, Mr. W. A. Maggs, F. 9 |
| Paddington Green Children's Hospital, Paddington | *Surgeon*, Mr. C. Vincent Cotterell, W. 9 |
| Royal Free Hospital, Gray's Inn Road, W.C. | *Surgeon*, Mr. H. Harris, M. Th. |
| Royal Hospital for Children and Women, Waterloo Bridge Road, S.E. | *Surgeon*, Mr. Whitehouse, F. 1.30 |
| St. Bartholomew's Hospital, Smithfield, E.C. | *Surgeons*, Messrs. Ewbank, Tu.; Paterson, F.; Ackery, F.; Mackrett, Tu. Hour, 9 |
| St. George's Hospital, Hyde Park Corner, S.W. | *Surgeon*, Mr. Winterbottom, Tu. S. 9 |
| St. Mary's Hospital, Cambridge Place, Paddington | *Susgeon*, Mr. H. Hayward, W. S. 9.30 |
| St. Thomas's Hospital, Westminster Bridge Road, S.E. | *Surgeon*, Mr. Truman, Tu. F. 10 |
| University College Hospital, Gower Street, W.C. | *Surgeon*, Mr. S. J. Hutchinson, W. 9.30 |
| Victoria Hospital for Children, Chelsea, S.W. | *Surgeon*, Mr. F. Fox, S. 9.0 |
| West London Hospital, Hammersmith Road, W. | *Surgeon*, Mr. H. L. Albert, Tu. F. 9.30 |
| Westminster Hospital, Broad Sanctuary, S.W. | *Surgeons*, Messrs. J. Walker and M. A. Smale, W. S. 9.15 |

### THROAT DISEASES.

| Hospitals and Terms of Admission. | Physicians, Surgeons, and Medical Officers, with Days and Hours of Attendance. |
|---|---|
| Central London Throat and Ear Hospital, Gray's Inn Road, W.C. | *Surgeons. See* "Ear Cases." M. W. Th. S. 2.30; Tu. F. 5 |
| Great Northern Central Hospital, Caledonian Rd, N. | *Surgeon*, Mr. Spencer Watson, W. 2 |
| Guy's Hospital, St. Thomas Street, S.E. | *Surgeon*, Mr. Symonds, Th. 1 |
| Hospital for Diseases of the Throat and Chest, 32, Golden Sq. Hon. Sec., Mr. G. C. Witherby. Poor free; others by small payment | *Physicians*, Drs. Wolfenden, Tu. F. 2.30; Macdonald, Tu. F. 6.30; Bond, W. S. 2.30<br>*Surgeon*, Mr. T. Mark Hovell, M. Th. 2.30 |
| King's College Hospital, Portugal Street, W.C. | *Surgeon*, Mr. Cartwright, Tu. F. 10 |
| Middlesex Hospital, Berners Street, W. | *Surgeon*, Mr. Hensman, Tu. 9 |
| North West London Hospital, 13, Kentish Tn. Rd., N.W | *Surgeon*, Mr. Collier, M. 1.30 |
| St. Bartholomew's Hospital, Smithfield, E.C. | *Surgeon*, Mr. Butlin, F. 2.30 |
| St. George's Hospital, Hyde Park Corner, S.W. | *Surgeon*, Dr. Whipham, Th. 1.30 |
| St. Mary's Hospital, Cambridge Place, Paddington | *Surgeon*, Mr. A. T. Norton, Tu. Fr. 1.30 |
| St. Thomas's Hospital Westminster Bridge Road, S.E. | *Physician*, Dr. Semon, Tu. F. 1.30 |
| University College Hospital, Gower Street, W.C. | *Physician*, Dr. Poore, Th. 1.30 |
| Westminster Hospital, Broad Sanctuary, S.W. | W. 9. *See* General Hospitals. |

### DISEASES OF WOMEN, (OBSTETRIC CASES, ETC.)

| Hospitals and Terms of Admission. | Physicians, Surgeons, and Medical Officers, with Days and Hours of Attendance. |
|---|---|
| Charing Cross Hospital, West Strand, W.C. | *Physicians*, Drs. J. W. Black, Amand Routh, Tu. F. 1.30 |
| Chelsea Hospital for Women, Fulham Road, S.W. Sec., Mr. J. H. Easterbrook. Payment from 10*s.* 6*d.* a week. Poor by Governor's letter. Out-patients, if without subscriber's letter, pay 6*d.* each attendance | *In-Physicians*, Drs. J. H. Aveling, M. Th.; A. W. Edis, Tu. F.; F. Barnes, W. S. Hour, 2.30<br>*Out-Physicians*, Drs. Dickinson and Mackeron, M.; W. Travers and G. Harper, Tu.; F. Jones and J. I. Parsons, W.; and H. T. Rutherford, F. Hour, 2 |

### DISEASES OF WOMEN, (OBSTETRIC CASES, ETC.)—*Continued.*

| Hospitals and Terms of Admission. | Physicians, Surgeons, and Medical Officers, with Days and Hours of Attendance. |
|---|---|
| East London Hospital for Children and Dispensary for Women, Shadwell, E. A Subscriber's letter is required, except in urgent cases | *Out-Physicians*, M. Tu. W. Th. F. 1 and 3; Tu. F. 9. *See* "Children's Diseases." |
| German Hospital, Dalston Lane, Dalston, E. | *Physician*, Dr. Rasch, M. Th. 9 |
| Great Northern Central Hospital, Caledonian Rd., N. | *Physician*, Dr. F. Barnes, W. 2 |
| Grosvenor Hospital for Women and Children, 3 and 4, Vincent Square, S.W. | *Out-Physicians and Surgeons*, Tu. W. Th. S. 2. *See* "Children's Diseases." |
| Guy's Hospital, St. Thomas Street, S.E. | *Physicians*, Drs. Galabin, Horrocks, M. Tu. F. 1.30 |
| Hospital of St. John and St. Elizabeth, 47, Gt. Ormond Street, W.C. For females suffering from long standing disease. | *Physicians*, Drs. Constable and Tegart (no out-patients) |
| Hospital for Women, 29 and 30, Soho Sq., W. Sec., Mr. David Cannon. In-patients admitted free by Governor's letter; also by payment. Out-patients free | *In-Physicians*, Drs. Carter, W. S. 2; R. T. Smith, Tu. F. 2; E. Holland, Tu. F. 9.30<br>*In-Surgeon*, Mr. H. A. Reeves, M. 4, Th. 2;<br>*Out-Physicians*, Drs. R. T. Smith, F. 10; E. Holland, W. 10; Mansell-Moullin, M. Th. 11; Bedford Fenwick, Tu. 10; J. Oliver, S. 10<br>*Out-Surgeon*, Mr. S. Osborn, Th. 2 |
| King's College Hospital, Portugal Street, W.C. | *Physician*, Dr. Playfair, Tu. Th. S. 2 |
| London Hospital, Whitechapel Road, E. | *In-Physician*, Dr. Herman, Tu. F. 1.30<br>*Out-Physician*, Dr. Lewers, W. S. 1.30 |
| Metropolitan Free Hospital, Gray's Inn Road, W.C. | *Physician*, Dr. A. Venn, W. 2 |
| Middlesex Hospital, Berners Street, W. | *In-Physician*, Dr. Edis, Tu. F. 1.30<br>*Out-Physician*, Dr. Duncan, W. S. 1.30 |
| New Hospital for Women, 222, Marylebone Road, W. Hon. Sec., Miss C. Vincent. By subscriber's letter. Outpatients pay 6*d.* entrance fee, and 2*d.* each visit. | *In-Physicians*, Drs. Anderson, Atkins, Marshall, de la Cherois<br>*Out-Physicians*, Drs. Marshall, De la Cherois, Hitchcock. Daily, 1 to 3, and S 9 to 10. (All the physicians are women) |
| North-West London Hospital, 18, Kentish Tn. Rd., N.W | W. 1.30. *See* General Hospitals |
| Royal Free Hospital, Gray's Inn Road, W.C. | *In- and Out-Physician*, Dr. Hayes, Tu. S. 9 |
| Royal Hospital for Children and Women, Waterloo Bridge Road, S.E. Sec., Mr. R. G. Kestin. | *Out-Physicians*, Daily at 2<br>*Out-Surgeon*, W. 2<br>*See* "Children's Diseases." |
| St. Bartholomew's Hospital, Smithfield, E.C. | *In-Physician*, Dr. M. Duncan, Tu. Th.<br>*Out-Physician*, Dr. Godson. W. S. 9 |
| St. George's Hospital, Hyde Park Corner, S.W. | *Physician*, Dr. Champneys, Th. 1.30 |
| St. Mary's Hospital, Cambridge Place, Paddington, W. | *Physicians*, Drs. Braxton Hicks, Tu. F. 9.30; H. Jones, M. Th. 1.30 |
| St. Thomas's Hospital, Westminster Bridge Road, S.E. | *Physician*, Dr. Jervis, Tu. F. 2 |
| Samaritan Free Hospital for Women and Children, Lower Seymour Street, W. Branch, 1, Dorset Street, Manchester Square, W. Sec., Mr. Geo. Scudamore. Governor's letter or card is required if the disease of the applicant is *not* peculiar to her sex. Out-patients department (free) is at Edward's Mews, Duke Street, W. | *In-Physicians*, Drs. P. Boulton, M Prickett, Amand Routh. Daily, 2<br>*In-Surgeons*, Messrs. G. G. Bantock, J. H. Thornton, W. A. Meredith. Daily, 9.30<br>*Out-Physicians*, Drs. M. Prickett, and Amand Routh, W. S. 1.30<br>*Out-Surgeons*, Messrs. W. A. Meredith, and J. D. Malcolm, M. Th.; A. H. G. Doran, and W. S. A. Griffith, Tu. F. Hour 1.30 |
| University College Hospital, Gower Street, W.C. | *Physicians*, Drs. G. Hewitt, M. Th. 1.30; J. Williams, Tu. F. 2 |
| West London Hospital, Hammersmith Road, W. | *Physicians*, Drs. A. Venn, Moullin, W S. 2 |
| Westminster Hospital, Broad Sanctuary, S.W. | *Physicians*, Drs. Potter, Tu. F. 2; Grigg, Tu. F. 9 |

### THE CHILDREN'S CORNER.

**Puzzles—Fifth Week.**

No. 17. GEOGRAPHICAL ACROSTIC. 1. A small singing-bird. 2. An exclamation. 3. A large island to the north of Australia. 4. A bank to confine water. 5. A foreign fruit. 6. A city in France. The initials name a large city in England, the finals the river on which it stands.

No. 18. ENIGMA. Take away my first letter, take away my second letter, take away all my letters, and I am still the same.

No. 19. ARITHMETICAL RIDDLE. A cat in each corner of the room; a cat sitting on each cat's tail; a cat sitting opposite to each cat. How many cats?

No. 20. I am all attention; transpose me, and I become dumb.

**Letters—Fifth Week.**

Next week write me an account of your favourite *Studies.*

**Results of Third Week—Puzzles.**

No. 9. BURIED COUNTIES AND TOWNS. 1. Norfolk. 2. Derby. 3. Waterford. 4. Bedford. (I am sorry there was a misprint in this puzzle. It should have read " Here is a bed for dolly," instead of a dolly.) 5. Cheshire.

No. 10. THE PROVERB, " All is not gold that glitters," was easily guessed by all.

No. 11. TREE PUZZLE. The tree which is nearest the sea is of course the beech (beach). The chronological tree is the date, and the one which is most warmly clad must be the fir (fur) tree.

No. 12. CHARADE. The winner of the fight is the " Victor," and now add *ia*, and you get the name of our own Queen, " Victoria."

*All right*—Le Cerf Agile, A. G. S. McCulloch, Mikado, Agatha, A. C. K., Umslopogaas, Alsie, Toby, The Eda, Amy, Mount Edgcumbe, Violin, Scrooge, Ethel; *Three right*—Skye, Ossian, Joe, Princess Ida, Poodle; *Two right*—Socrates.

**Letters.**

BOATING.

The best letters this week have naturally come from boys. All children are not fortunate enough to live near the sea or a river, but those who are, have written interesting accounts of their boating experiences. I have only space for three letters. Here is an exciting account of the adventures of The Eda:—

We are very fond of boating indeed; but three weeks ago, papa took us and some friends for a row in the evening in the bay. It was quite warm and fine when we set out, and we rowed easily to Saltcoats; but, coming back, the wind got up, and blew very hard against us. There was a beacon-light near us, and we looked every now and then to see if we were getting nearer home, but we seemed as if we never got any nearer, only in front of it. So we called and shrieked for the boatman to send out a boat to tug us in; but no one heard. At last the tug came out to tug in a big ship to the harbour. It saw us, and when it got back, it sent out a boat and some men, but they had to get to us first, and the time seemed so long, for it had got very cold and dark too, and the waves were splashing into the boat, and all of us were as wet and cold as possible. It was long past ten before we got home. Mamma and everybody were so glad to see us, as the rocks are so dangerous round the coast, and the current so strong when the wind is blowing; and we were glad to be at home, and warm by the fire; and bed was a capital place to go to, after the boat, and the wind, and the waves.

Here is a letter from a Naval Officer's son (Toby, aged 14):—

When I lived at Portsmouth, as my father was an officer in the Navy, we had a house in the dockyard, and many were the enjoyable afternoons that I, in common with others, spent. On one occasion we borrowed a large dingy, took our dinner, and went out for a regular spree. We started about ten o'clock in the morning, and set up an improvised sail, as there was a strong wind blowing, which carried us out of the boat-pond in which we were, and through the mouth of the harbour. We then wore round, dropped our anchor, and commenced to fish. At first we caught nothing, but at last we hooked some whiting. One of these was pulled up to the very top of the water, when the hook slipped, and the boy who had hooked the fish, grabbing at it, overbalanced himself, and went a header overboard. We caught him by the leg, however, and managed to haul him in, very nearly wet through. We thought that we would then adjourn for dinner, but we found that in pulling him in we had shipped so much water, that all our provisions were soaked with salt water, so that they were not eatable. We then pulled up our anchor, turned round, and began to pull back. Owing to the wind and tide being against us, we found this a rather difficult job. However, after great exertions, in which we broke one of the pair of sculls that had been lent us, we reached our starting-place about two o'clock, ravenously hungry and very wet. This is one of my experiences of boating on the sea.

Socrates (aged 13) writes a good letter:—

DEAR SIR,—Boating and fishing are about the jolliest things out, I think. I like boating more on rivers than on the sea, as it is generally prettier, although I must own that rowing near the sea-coast is sometimes very beautiful. The seaweed-covered rocks, and little caves and recesses, all together form a very pretty picture. Last time I went on the river was about a fortnight ago. We rowed up to Hampton Court, and after getting out of the boat to have luncheon, and enjoy the beautiful scenery which lay spread before us, we rowed back again all together, having had a very enjoyable day. Boating also does one good, it makes you stronger and more healthy. My cousin had a fine little yacht, a racing one, and the other day was racing with a friend of his who also has one. Just as they got to the most critical part of the race, a steam yacht ran right into my cousin's, and split it in two. It went down in twenty seconds, but happily everyone was saved.

One mark each:—The Eda, Toby, Socrates, Skye, Ossian, Le Cerf Agile, Mikado, A. G. S. McCulloch, Mount Edgcumbe, Princess Ida, Ethel.

**Notices to Correspondents.**

AMY and ETHEL.—Only one set of answers can be accepted, unless two coupons are sent.

OSSIAN.—Your letters please me very much.

### AMUSEMENTS WITH PROFIT.

**No. 3. Double Acrostic.**

Our gallant soldier-citizens, with pomp and pride of war,
To a peaceful work of conquest came marching from afar;
They charge the well-spread tables and fall upon the foe,
With prowess that gives earnest of the deeds that they can do.

1. Dwelling-place of gods and heroes in Scandinavian lore;
2. Pleasant 'tis to rest upon it ere we reach the distant shore;
3. Seek not, mortal, to escape it, 'tis assigned thee from on high;
4. Home of gallant mountain patriots who fought for liberty;
5. Least orthodox of bishops; day to children ever dear;
6. Forth sprung his fatal arrow as the hated king drew near;
7. Without this the brightest flowers and the fairest scenes on earth;
8. Without that all sweetest melodies were found of little worth;
9. Trust not Fancy's bright illusions, trust not ev'ry fleeting dream;

For life, we know, is earnest, and " things are not what they seem."

**No. 4. Short Essay.**

Write a short essay on the " Power of Association."

**Answers.**

No. 25. DOUBLE ACROSTIC.

P ai R
A rn O
G u Y
E r A
A ppea L
N ough T
T or Y

Correct answers have been received from Gretta, Dawnlover, Aralc.

No. 26. ALGEBRAIC PROBLEM.

Let $x$ and $y$ = digits
Then No. is $10x + y$

$$(\alpha)\quad \frac{10x+y}{x+y} = x \quad \therefore \quad 10x + y = x^2 + xy$$

$$(\beta)\quad \frac{10y+x}{x+y} = 7 \quad \therefore \quad 10y + x = 7x + 7y$$

$$-6x = -3y$$

$$\therefore y = 2x$$

By substitution $(\alpha)$ $10x + 2x = x^2 + 2x^2$

$$\therefore 3x^2 = 12x$$

$$x = 4$$

And $\therefore$ $y = 2 \times 4 = 8$

$\therefore$ No. is 48.—*Ans.*

Other solutions have been received, some of which are quite impossible.

Correct answers have been received from Common Sense, Aralc, Boss, Gretta, Letheàdor, Mater, Dawnlover.

N B.—All answers to the problems must be accompanied by the workings.

**Results of Second Quarterly Competition.**

" Dawnlover" and " Gretta" have each gained twenty-four correct answers. We therefore propose, as before, either (1) to divide the prize, leaving both competitors still free to enter again for the Three Guinea Prize; or (2) to set a test acrostic to decide the tie. We shall be glad to hear from " Dawnlover" and " Gretta" which course they prefer.

